# Effects of resveratrol on biochemical and structural outcomes in osteoarthritis: A systematic review and meta-analysis of preclinical studies

**DOI:** 10.1016/j.heliyon.2024.e34064

**Published:** 2024-07-04

**Authors:** Wenjian Zhao, Yuezhi Zhu, Sok Kuan Wong, Norliza Muhammad, Kok-Lun Pang, Kok-Yong Chin

**Affiliations:** a Department of Pharmacology, Faculty of Medicine, Universiti Kebangsaan Malaysia, 56000, Cheras, Malaysia; b Department of Pathology, College of Basic Medicine, Xiangnan University, 423000, Chenzhou City, China; c Department of Biochemistry, Faculty of Medicine, Universiti Kebangsaan Malaysia, 56000, Cheras, Malaysia; d Newcastle University Medicine Malaysia, 79200, Iskandar Puteri, Malaysia

**Keywords:** Arthritis, Chondrocytes, Joint, Stilbenoid, Subchondral bone

## Abstract

**Background and objective:**

Osteoarthritis (OA) is the most common age-related disease of joints with increasing global prevalence. Persistent inflammation within the joint space is speculated to be the cause of OA. Resveratrol is an anti-inflammatory and antioxidant compound which can influence cartilage metabolism through multiple signalling pathways. This systematic review and meta-analysis aimed to summarize the therapeutic effects of resveratrol in animal models of OA.

**Methods:**

A comprehensive literature search was performed using PubMed, Embase, Web of Science, Cochrane Library, China National Knowledge Infrastructure, China Wanfang and VIP databases in May 2023. Studies on the effects of resveratrol in animal models of OA written in English or Mandarin, published from the inception of databases until the date of the search were considered.

**Results:**

Fifteen eligibility studies were included and analysed. Resveratrol was shown to inhibit the secretion of interleukin-1β, tumour necrosis factor-α, interleukin-6, nitric oxide, and apoptosis of articular chondrocytes. Joint structure as indicated by Mankin scores was restored with resveratrol in animal OA models.

**Conclusion:**

Resveratrol is a potential therapeutic agent for OA based on animal studies. Further evidence from well-planned human studies would be required to validate its clinical efficacies.

## Highlights

• Resveratrol suppresses joint inflammation, marked by reduced pro-inflammatory markers, in animal models of osteoarthritis.
• Resveratrol reduces chondrocyte apoptosis and improves cartilage structure in animal models of osteoarthritis.
• The promising effects of resveratrol warrant a well-planned clinical trial to validate its anti-arthritic activities.

## 1 Introduction

Osteoarthritis (OA) is the most common age-related arthropathy, characterised by the degradation of the articular cartilage with degenerative changes of other joint components, such as bone, meniscus, and synovium [1]. Primary OA refers to the degeneration of articular cartilage without strong underlying factors, while secondary OA is due to consequences of specific causes such as joint trauma, infection and other abnormalities [2,3]. The presence of these primary and secondary factors individually or synergistically will eventually lead to OA [4]. Imaging features of OA, such as narrowing of the joint space, osteophytes, irregularity of the joint surface, and cartilage damage, are mainly seen in people over 65 years of age, and about 80 % of people over 75 years of age suffer from OA [5,6]. As global ageing continues, patients with OA are also increasing. This can cause severe disability and affect the productive life of patients and impose a heavy burden on medical care [[7], [8], [9]].

Although OA is traditionally considered a non-inflammatory disease, the current research shows that inflammation contributes to the development of the disease [[10], [11], [12]]. In OA, synovium develops an inflammatory response and releases pro-inflammatory substances such as cytokines and chemokines. These pro-inflammatory mediators cause lateral damage to joint tissues, leading to pain, stiffness, and swelling of the joints [4,12]. Inflammatory mediators also trigger the production of matrix metalloproteinases (MMPs), which accelerate the breakdown of the cartilage matrix, resulting in the loss of cartilage integrity and the development of OA [2].

Standard OA management includes non-pharmacological, pharmacological and surgical treatments [[13], [14], [15]]. Non-pharmacological treatments include weight control, physical therapy, and lifestyle changes. Medications such as symptomatic slow-acting drugs for OA, non-steroidal anti-inflammatory drugs (NSAIDs), and analgesics are often prescribed for symptomatic patients [[13], [14], [15]]. However, long-term use of these drugs like NSAIDs has many adverse effects, such as increased risk of gastrointestinal bleeding and toxicity to kidneys, liver and cardiovascular systems [[16], [17], [18], [19]]. Surgical intervention may be considered when other treatment modalities are ineffective in relieving symptoms [8]. Standard surgical options include arthroscopic surgery, joint replacement surgery (e.g., knee replacement or hip replacement), and arthrocentesis [8]. Surgical treatments are invasive and costly [9]. Additionally, arthroscopy only relieves the pain transiently and does not improve the clinical outcomes of OA [20,21]. Therefore, the search for an effective and safe treatment that would slow down or stop the progression of OA is ongoing.

Resveratrol (*trans*-3,4′,5-trihydroxystilbene) is a bioactive compound commonly found in several plants like grapevine (*Vitis vinifera*), knotweed (*Polygonum cuspidatum*) and *Veratrum grandiflorum* [22]. Resveratrol has demonstrated anti-inflammatory, antioxidant and anti-tumour effects in previous studies [[22], [23], [24]]. In addition, various studies have reported the therapeutic effects of resveratrol against chronic or age-related diseases [25,26]. Resveratrol can improve articular cartilage damage by antagonising the production of cartilage-degrading proteases [27,28]. Additionally, resveratrol also activates Sirtuin 1 (SIRT1) signalling pathway and reduces the secretion of inflammatory cytokines through various signalling pathways, thus preventing the vicious intra-articular inflammation in OA [29,30]. These properties of resveratrol could be harnessed to protect the joint against OA. Several animal studies have examined the effects of resveratrol on OA. Particularly, doubt remains as resveratrol could penetrate the avascular joint space to suppress local inflammation in OA and improve the chondrocyte's survival.

To the best of our knowledge, there is a lack of systematic review on the joint benefits of resveratrol in animal studies of OA. Therefore, this systematic review and meta-analysis aim to summarize and analyse the current evidence on the effects of resveratrol on the progression of OA using animal models, particularly on joint inflammation, cartilage changes and chondrocyte apoptosis. The evaluation of the efficacy of resveratrol in animal models of OA can provide a reference for subsequent relevant clinical research.

## 2 Materials and methods

This systematic review and meta-analysis were written based on the Preferred Reporting Items for Systematic Reviews and Meta-Analyses (PRISMA) [31]. The protocol has been registered in Open Science Framework (url: https://osf.io/fndvj/).

### 2.1 Literature search

A literature search was performed using PubMed, Embase, Web of Science, Cochrane Library, China National Knowledge Infrastructure, China Wanfang and VIP databases in May 2023. All preclinical studies on the effects of resveratrol on OA were considered. A literature search was performed using the English search string: resveratrol AND osteoarthritis, or the Chinese search string: "骨关节炎" AND "白藜芦醇".

### 2.2 Inclusion and exclusion criteria

Inclusion criteria were [1] primary studies using animal models of OA [2]; interventions using resveratrol [3]; articles written in English or Chinese.

Exclusion criteria were [1] lack of assessment of resveratrol's effects on the joint [2]; experimental studies without negative or OA controls [3]; studies that use a combination of resveratrol with other agents [4]; articles without primary data (reviews, letters and perspectives) [5]; conference abstracts due to incomplete data or potential overlapping with research articles.

### 2.3 Screening of literature

Three investigators (W.Z., Y.Z. and K.Y.C.) screened titles and abstracts of the retrieved literature for relevant studies. The full text of potential articles was obtained and screened according to the inclusion and exclusion criteria. Any discrepancy among the researchers is resolved based on discussion.

### 2.4 Data extraction

After screening the literature, the data were extracted by two investigators (W.Z. and Y.Z.) independently. The data extracted included the authors/year, country, characteristics of the animals used, induction of OA, sample size, intervention type and time, as well as outcomes measured etc. Any controversies during the data extraction process were resolved by consulting the corresponding author (K.Y.C.).

### 2.5 Risk of bias assessment

The risk of bias assessment was performed using the Systematic Review Centre for Laboratory Animal Experimentation (SYRCLE) risk of bias tool for animal studies [32]. It covers random sequence generation, allocation concealment, random housing, blinding, random outcome assessment, missing data reporting, selective outcome reporting and other sources of biases [32]. Two assessors (W.Z. and Y.Z.) ranked each item low, high or unclear risk. Any discrepancies were resolved by consulting the corresponding author (K.Y.C.).

### 2.6 Statistical analysis

The study was statistically analysed using Review Manager (RevMan) Version 5.4 [33]. Mean difference (MD) or standardised mean difference (SMD) and 95 % confidence interval (CI) were used as effect indicators. I^2^ statistics assessed the heterogeneity and inconsistency between the included literature outcome variables. Meta-analysis was performed using the fixed-effects model if I^2^<50 %, and conversely, the random-effects model. Publication bias between included literature was evaluated using the funnel plot [34,35]. Power analysis was performed using R version 4.4.0, (The R Foundation for Statistical Computing) with the package “metapower” (https://cran.r-project.org/package=metapower) [36].

## 3 Results

### 3.1 Study search results

**Fig. 1:**
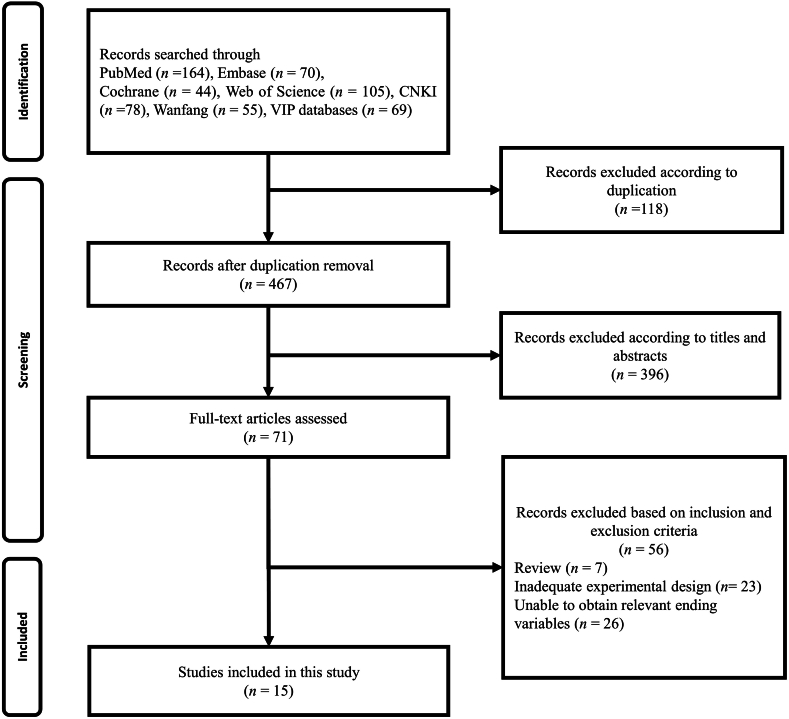
PRISMA flowchart summarising the process of study identification, screening and inclusion.

A total of 585 relevant papers were retrieved from the literature search. During the title and abstract screening, 570 were eliminated due to various reasons (26 articles do not contain primary data; 544 articles do not fulfil the criteria of the review.). Finally, the full text of 15 articles was retrieved and included in the final analysis [[37], [38], [39], [40], [41], [42], [43], [44], [45], [46], [47], [48], [49], [50], [51]]. The literature screening process and results are shown in Fig. 1.

### 3.2 Basic characteristics of the studies and quality evaluation

**Fig. 2:**
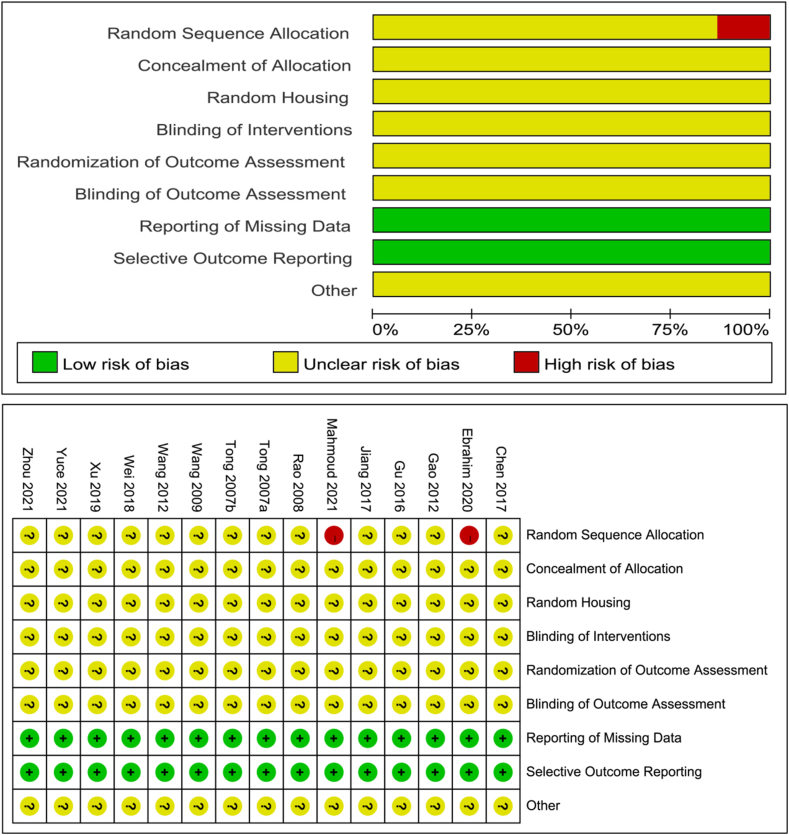
Risk of bias analysis was performed on the 15 included research studies with the SYRCLE tool. The low risk of bias is presented in green, the unclear risk is presented in orange, and the high risk of bias is presented in red. (For interpretation of the references to colour in this figure legend, the reader is referred to the Web version of this article.)

The characteristics of the literature included in this study are shown in Table 1. A total of 262 animals were used in the 15 studies included in this review, wherein 131 were in the experimental group and 131 were in the model group. The studies were published by research groups from China, Turkey, and Saudi Arabia. The experimental animals were rabbits, mice and rats. OA was induced by Hulth methods in rabbits, high-fat diet in mice, monosodium iodoacetate or destabilization of medial meniscus in rats. Only one study examined the effects of resveratrol on temporomandibular joint OA in rats [38]. The resveratrol treatment time ranged from 2 to 12 weeks. The dose of resveratrol varies according to the types of animals used and the routes of administration. The route of administration was either oral gavage or intra-articular injection. Risk of bias assessment based on the SYRCLE tool revealed that no selective reporting of results in all studies. However, two studies did not indicate randomisation of groups [43,45]. All studies did not mention blinding and allocation concealment. The specific quality evaluation results are shown in Fig. 2.

**Table 1:** Basic characteristics of the study.

Included studies (author and year)	Country	Experimental animal characteristics	Age	Induction of OA	Sample size (n)		Intervention time	Intervention measures		Outcome
					Experimental group	Control group		Experimental group	Control group	
Tong et al., 2007a [46]	China	New Zealand rabbits (male:female 1:1)	N/A	Hulth	6	6	6 weeks	Resveratrol 100 mg/kg/day (per os)	Sanitary saline	⑤
Tong et al., 2007b [50]	China	New Zealand rabbits (male:female 1:1)	N/A	Hulth	6	6	4 weeks	Resveratrol 120 mg/kg/day (per os)	Sanitary saline	⑤
Rao et al., 2008 [51]	China	New Zealand rabbits (male:female 1:1)	7 months	Hulth	6	6	45 days	Resveratrol 120 mg/kg/day (per os)	Sanitary saline	⑥
Wang et al., 2009 [47]	China	New Zealand rabbits (male:female 1:1)	7 months	Hulth	6	6	6 weeks	Resveratrol 120 mg/kg/day (per os)	Distilled water	②
Wang et al., 2012 [37]	China	New Zealand rabbits (male:female 1:1)	N/A	Hulth	6	6	2 weeks	Resveratrol 50 μmol/kg/day (intra-articular)	Dimethyl sulfoxide	②⑤
Gao et al., 2012 [48]	China	New Zealand rabbits (male:female 1:1)	N/A	Hulth	6	6	6 weeks	Resveratrol 120 mg/kg/day (per os)	Sanitary saline	③
Gu et al., 2016 [40]	China	C57BL/6 J mice (male)	7 weeks	High-fat diet	12	12	12 weeks	Resveratrol 45 mg/kg/day (per os)	Blank control	①
Jiang et al., 2017 [39]	China	C57BL/6 J mice (male)	7 weeks	High-fat diet	15	15	12 weeks	Resveratrol 45 mg/kg/day (per os)	Blank control	①③
Chen et al., 2017 [49]	China	New Zealand rabbits (male:female 1:1)	4 months	Hulth	8	8	6 weeks	Resveratrol 50 μmol/kg/day (intra-articular)	Dimethyl sulfoxide	①
Wei et al., 2018 [42]	China	Wistar rats (male)	6 weeks	Monosodium iodoacetate	10	10	8 weeks	Resveratrol 50 mg/kg/3day (per os)	Distilled water	③④⑥
Xu et al., 2019 [44]	China	C57BL/6 J mice (male)	7 weeks	High-fat diet	13	13	8 weeks	Resveratrol 45 mg/kg/day (per os)	Blank control	①
Ebrahim et al., 2020 [45]	Saudi Arabia	Albino rats (male)	N/A	High-fat diet	8	8	12 weeks	Resveratrol 30 mg/kg/day (per os)	Blank control	①④⑥
El-Bidawy et al., 2021 [43]	Saudi Arabia	Albino rats (male)	10 weeks	High-fat diet	8	8	12 weeks	Resveratrol 30 mg/kg/day (per os)	Blank control	④⑥
Yuce et al., 2021 [38]	Turkey	Wistar albino rats (male)	N/A	Temporomandibular joint OA	8	8	4 weeks	Resveratrol 1 mg/80 μL/3 times a week (intra-articular)	Dimethyl sulfoxide	②
Zhou et al., 2021 [41]	China	New Zealand rabbits (male)	3 months	Destabilization of medial meniscus	8	8	2 weeks	Resveratrol 15 μmol/L/2 days (intra-articular)	Sanitary saline	①

Outcome indicators: ① Mankin grading system score; ② Chondrocyte apoptosis positive expression rate; ③ Interleukin-1β level; ④ Tumour necrosis alpha-α level; ⑤ Nitric oxide level ⑥ Interleukin-6 level.

### 3.3 Meta-analysis results

#### 3.3.1 Interleukin (IL)-1β level

**Fig. 3:**
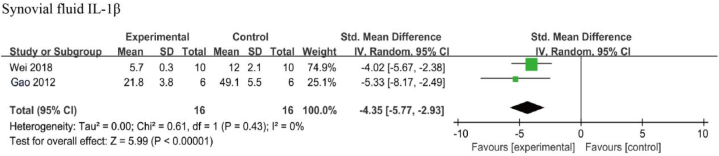
Synovial IL-1β level in animals with OA treated with resveratrol. The combined results showed that resveratrol reduced synovial IL-1β levels significantly.

Three studies examined the effects of resveratrol on IL-1β levels, two of which investigated its synovial levels [42,48] while another investigated its serum level [39]. As there was only one study on serum IL-1β levels, the meta-analysis was not performed. A negligible heterogeneity was found among the included literature (I^2^ = 0 %, *p* > 0.05), thus a fixed-effects model was used for the meta-analysis. The results suggested that IL-1β levels in the synovial fluid [SMD: −4.35, 95 % CI (−5.77, −2.93), *p* < 0.01] were significantly lower than the negative control group (Fig. 3).

#### 3.3.2 TNF-α level

**Fig. 4:**
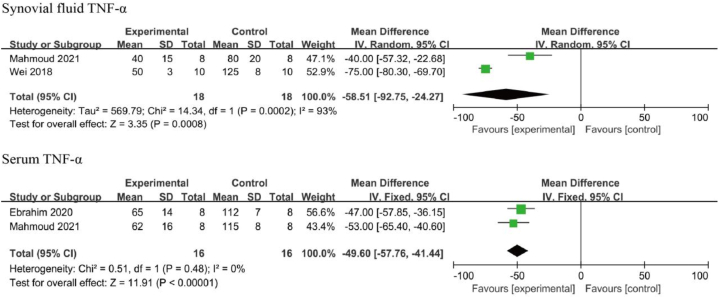
TNF-α level in animals with OA treated with resveratrol. The combined results showed that resveratrol reduced both synovial and serum TNF-α levels.

Two studies examined the effects of resveratrol on synovial TNF**-**α levels [42,43], and two studies examined the effects of resveratrol on serum TNF**-**α levels [43,45]. The heterogeneity test suggested considerable heterogeneity between the two included studies on TNF**-**α levels in synovial fluid (I^2^ = 93 %, *p* < 0.05), thus a random-effects model was used for this analysis. A negligible heterogeneity was found between the two included studies on serum TNF**-**α levels (I^2^ = 0 %, *p* = 0.48), thus a fixed-effects model was used for this analysis. Subgroup analysis according to the sampling sites revealed that TNF-α levels in the synovial fluid [MD: −58.51 95 % CI (−92.75, −24.27), *p* < 0.01] and serum [MD: −49.60, 95 % CI (−57.76, −41.44), *p* < 0.01] of the resveratrol-treated group were significantly lower than the control group (Fig. 4).

#### 3.3.3 IL-6 level

**Fig. 5:**
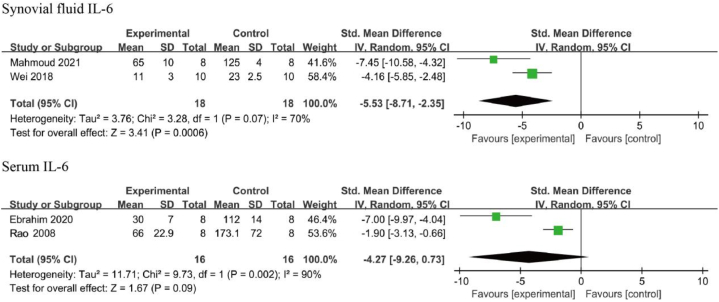
IL-6 level in animals with OA treated with resveratrol. The combined results showed that resveratrol reduced synovial IL-6 levels significantly, but not serum IL-6 levels.

Two studies examined the effects of resveratrol on synovial IL-6 levels [42,43], and two studies examined the effects of resveratrol on serum IL-6 levels [45,51]. A considerable heterogeneity was found among the included studies on synovial fluid (I^2^ = 70 %, *p* < 0.01) and serum (I^2^ = 90 %, *p* < 0.01), thus a random-effects model was used to perform this analysis. IL-6 levels in the synovial fluid of the resveratrol-treated group [SMD: −5.53, 95 % CI (−8.71, −2.35), *p* < 0.01] were significantly lower than that of the negative control group. However, the serum IL-6 levels were not statistically significant between the two groups [SMD: −4.27, 95 % CI (−9.26, 0.73), *p* = 0.09] (Fig. 5).

#### 3.3.4 Nitric oxide (NO) level

**Fig. 6:**
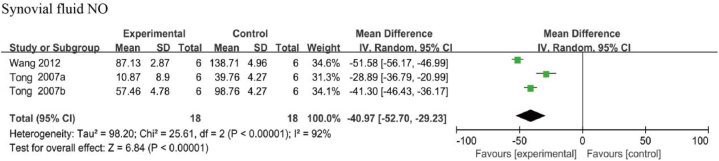
Synovial NO level in animals with OA treated with resveratrol. The combined results NO levels in the synovial fluid were significantly lower than the negative control group.

Four studies examined the effects of resveratrol on NO levels, whereby three examined its synovial levels [37,46,50] and one examined its serum level [50]. As there was only one study on serum NO levels, a meta-analysis was not performed. The three studies on synovial NO levels reported a considerable heterogeneity (I^2^ = 92 %, *p* < 0.01), so a random-effects model was used for the analysis. The analysis showed that NO levels in the synovial fluid were significantly lower than the negative control group [MD: −40.97, 95 % CI (−52.70, −29.23), *p* < 0.001] (Fig. 6).

#### 3.3.5 Chondrocyte apoptosis

**Fig. 7:**
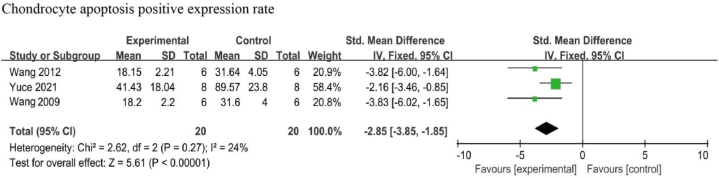
Chondrocyte apoptosis in animals with OA treated with resveratrol. The combined results showed that the chondrocyte apoptosis in the resveratrol-treated group was significantly lower than the negative control group.

Three studies examined the effects of resveratrol on chondrocyte apoptosis [37,38,47]. A negligible heterogeneity was reported among the included studies (I^2^ = 24 %, *p* = 0.27), thus a fixed-effects model was used for this analysis. The combined results showed that the chondrocyte apoptosis in the resveratrol-treated group was significantly lower than the negative control group [SMD: −2.85, 95 % CI (−3.85, −1.85), *p* < 0.001] (Fig. 7).

#### 3.3.6 Mankin scores for cartilage structure

**Fig. 8:**
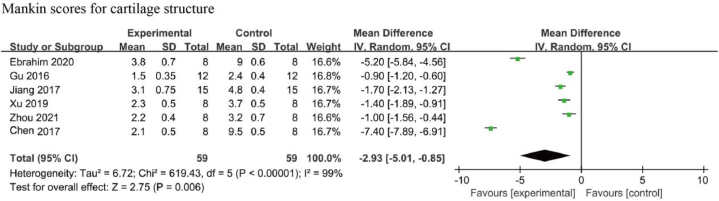
Mankin scores for cartilage structure in animals with OA treated with resveratrol. The combined results showed that the Mankin scores were significantly lower in the resveratrol-treated group than the negative control group.

Six studies examined the effects of resveratrol on Mankin scores for cartilage structure [[39], [40], [41],^44^,^45^,^49^]. A significant heterogeneity was reported among the included studies (I^2^ = 99 %, *p* < 0.01), thus a random-effects model was used for this analysis. The combined results showed that the Mankin scores were significantly lower in the resveratrol-treated group than the negative control group [MD: −2.93, 95 % CI (−5.01, −0.85), *p* < 0.01] (Fig. 8).

#### 3.3.7 Publication bias

**Fig. 9:**
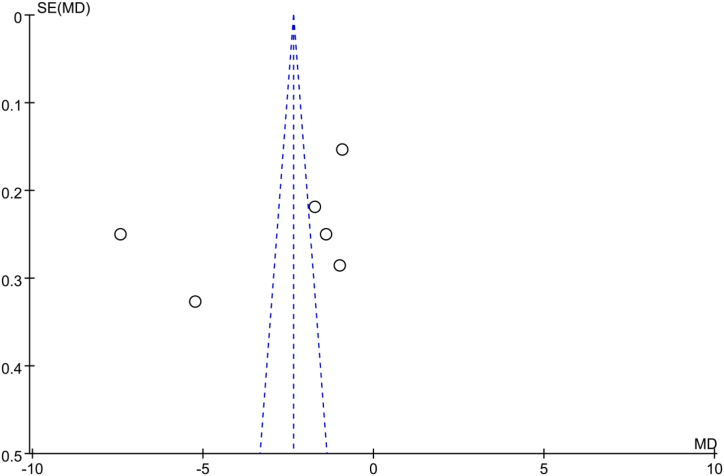
Publication bias for Makin scores. It is asymmetrical and indicates potential publication bias.

The funnel plot was used to test for publication bias in the Mankin scores. The plot showed that the scattered points were equally distributed outside the funnel plot, and the graph was asymmetrical (Fig. 9), indicating potential publication bias.

#### 3.3.8 Power analysis

Power analysis was performed for all the indices analyses. All indices except synovial TNF-a, serum IL-6 and Mankin scores achieved satisfactory power (>0.8) (Table 2).

**Table 2:** Power analysis for each studied index.

Parameters	Effect size	Sample size	Number of groups	I^2^	Calculated power
Synovial IL-1	4.35	16	2	0	1.000
Synovial TNF- α	58.51	18	2	0.93	0.691
Serum TNF-α	49.6	16	2	0	1.000
Synovial IL-6	5.53	18	2	0.7	0.94
Serum IL-6	4.27	16	2	0.9	0.658
Synovial NO	40.97	18	3	0.92	0.814
Chondrocyte apoptosis	2.85	20	3	0.24	1.000
Mankin score	2.93	59	6	0.99	0.521

#### 3.3.9 Sensitivity analysis of Mankin scores

A sensitivity analysis was performed using a study-by-study exclusion approach for individual studies. The results showed no change in heterogeneity, and the combined effect remained statistically significant, indicating robust results.

## 4 Discussion

This systematic review identified 15 preclinical studies assessing the efficacy of resveratrol in the treatment of OA. Despite some heterogeneity in design characteristics, the results revealed that all studies reported significant protective effects of resveratrol on the disease progression of OA, especially in terms of the anti-inflammatory, prevention of chondrocyte apoptosis, and improvement in Mankin scores for joint structure. The meta-analysis performed on these articles showed that resveratrol significantly improved joint structure by inhibiting the release of inflammatory mediators (IL-1β, TNF-α and IL-6) and NO [an inflammatory mediator produced by inducible NO synthase (NOS)] and inhibiting chondrocyte apoptosis. However, most animal studies were conducted in male mice/rabbits, thus preventing the exploration of sexual differences in the joint skeletal effects of resveratrol. This research gap needs to be bridged in future studies since elderly women are disproportionately affected by OA [52].

Pro-inflammatory cytokines are a series of proteins that cause inflammation by affecting the activity, differentiation, and proliferation of immune cells in the organisms [11,53]. Pro-inflammatory cytokines such as IL-1β, TNF-α, IL-6 and IL-11 are secreted in response to stimuli and play a pivotal role in the acute inflammatory response by binding to specific receptors on the surface of target cells [54,55]. This binding initiates a cascade of molecular reactions within the target cells, triggering the expression of various genes and signalling pathways that promote inflammation [53,56]. IL-1β is one of the most prominent inflammatory cytokines in chronic degenerative joint diseases such as OA. IL-1β promotes chondrocyte apoptosis, which in turn leads to cartilage matrix destruction [57]. The action of IL-1β was mediated through the stimulation of NO production by endothelial cells, decreasing the proteins in the cartilage matrix polysaccharides, leading to cartilage matrix destruction [57,58]. Wei et al. showed that resveratrol (50 mg/kg/3 d for eight weeks) significantly lowered the IL-1β levels in the knee synovial fluid of OA rats [42]. Gao et al. showed that resveratrol (30, 60 and 120 mg/kg/d for six weeks) dose-dependently lowered IL-1β levels in the knee synovial fluid of OA rabbits [48]. Jiang et al. reported that serum IL-1β levels were reduced in OA mice after receiving oral supplementation of resveratrol (22.5 mg/kg/d or 45 mg/kg/d for 12 weeks) [39]. The higher dose of resveratrol (45 mg/kg/d) exerted better effects in suppressing IL-1β levels. Resveratrol regulates IL-1β expression potentially through Toll-like receptor 4/TNF receptor-associated factor 6 signalling pathways [59,60]. The meta-analysis of this study confirmed that resveratrol suppressed the inflammatory response in animals with OA by inhibiting IL-1β secretion in the serum and knee synovial fluid.

Multiple studies have demonstrated the involvement of the Janus kinase/signal transducers and activators of the transcription (STAT) signalling pathway in OA development, and IL-6 has been identified as a critical regulator of this pathway [[61], [62], [63]]. Specifically, IL-6 can interact with STAT2 to modulate the function of STAT3, which can, in turn, affect cellular processes such as proliferation and apoptosis [64]. In addition, IL-6 has been shown to have a catabolic effect on articular cartilage, impeding anabolism. Conversely, inhibiting IL-6 has been found to limit extracellular matrix remodelling and bone loss, thus preserving joint tissue homeostasis [53]. Rao et al. showed that serum IL-6 was dose-dependently suppressed in OA rabbits after resveratrol supplementation (30 mg/kg/d or 60 mg/kg/d or 120 mg/kg/d for 45 days) than the untreated rabbits with OA [51]. Ebrahim et al. showed significant suppression of serum IL-6 secretion in T2DM rats after resveratrol administration [45]. El-Bidawy et al. reported that IL-6 levels in knee synovial fluid of T2DM rats given resveratrol were significantly lower than that of untreated T2DM rats [43]. The meta-analysis confirmed that resveratrol was effective in inhibiting IL-6 levels in the knee synovial fluid.

TNF-α increased matrix metalloproteinase (MMP) activities in the ECM of the joint, thus promoting articular cartilage destruction [65,66]. Besides, it has been shown that TNF-α can act directly on the receptors on the surface of osteoclasts and activate downstream signalling pathways in osteoclasts, thus promoting osteoclast proliferation and activation. It also can interact with other inflammatory mediators to create an inflammatory environment that leads to persistent chronic inflammation in OA [67]. El-Bidawy et al. showed that TNF-α levels in the serum and knee synovial fluid were significantly lower in T2DM rats given resveratrol (30 mg/kg/d for 12 weeks) than in untreated rats [43]. Ebrahim et al. also showed that serum TNF-α levels were significantly suppressed in T2DM rats after oral administration of resveratrol (30 mg/kg/d for 12 weeks) [45]. Wei et al. showed that the TNF-α levels in the knee synovial fluid of OA rats decreased after resveratrol (50 mg/kg/3 d for eight weeks) supplementation [42]. The meta-analysis confirmed that resveratrol was effective in inhibiting TNF-α levels in both serum and knee synovial fluid in animals with OA.

NO plays various critical physiological roles in the body [43, 44]. It promotes the development of OA by triggering inflammatory responses and increasing the synthesis of inflammatory cytokines [45, 46]. NO can also cause oxidative damage and chondrocyte apoptosis by participating in apoptosis [46, 47]. NOS is a biological enzyme that plays a crucial role in the synthesis of NO. NOS can be categorised into three distinct subtypes: endothelial NOS (eNOS), neuronal NOS (nNOS), and inducible NOS (iNOS), each with their unique physiological functions and distribution in different tissues [46–48]. eNOS and nNOS are constantly expressed, while iNOS is inducible. iNOS is upregulated during inflammation as it was driven by inflammatory cytokines such as TNF-α, IL-6, and other factors such as bacterial toxoids [68,69]. Nevertheless, iNOS is a double-edged sword where it produces NO to kill microorganisms under physiological conditions, but prolonged and high NO production is known to cause excessive inflammation, neuropathic pain, diabetes, sepsis and OA [69,70]. Clinically, NO and iNOS levels were significantly increased in synovium and chondrocytes of patients with OA [70,71]. Continuous release of inflammatory mediators such as IL-1 and TNF-α by OA chondrocytes results in the upregulation of iNOS and excessive NO production, this leads to the persistent release of inflammatory cytokines and other catabolic processes, resulting in continuous damage to the synovial membrane and chondrocytes, ultimately leading to apoptosis [71].

The study by Wang et al. showed that NO in the serum and knee synovial fluid of OA rabbits was reduced dose-dependently after the intervention of resveratrol (10, 20 or 50 μmol/kg/d for two consecutive weeks) [37]. Tong et al. found that NO secretion in the knee synovial fluid was significantly inhibited after OA rabbits were given resveratrol (100 mg/kg/d for six consecutive weeks) [46]. The same research team subsequently administered resveratrol (120 mg/kg/d for four consecutive weeks) to OA rabbits and found that NO secretion in serum and knee synovial fluid was significantly inhibited [50]. The meta-analysis of this study found that resveratrol could inhibit NO secretion in serum and knee synovial fluid of animals with OA. Resveratrol has been demonstrated in studies to inhibit the expression of pro-inflammatory mediators IL-8 and iNOS through activation of the nuclear factor erythroid 2–related factor 2/Heme oxygenase-1 pathway, leading to a reduction in the inflammatory response [28,72]. In addition, resveratrol through SIRT1 overexpression can increase eNOS expression to affect endothelial cell function, thereby impeding atherogenesis [51, 52]. Thus, resveratrol positively affects the prevention and/or treatment of OA by modulating NO. However, it involves multiple signal transduction pathways that still need to be investigated in more depth.

As the primary cell type of cartilage tissue, chondrocytes play a crucial role in maintaining its structural and functional integrity. Chondrocyte dysfunction is a significant factor in the pathophysiology of OA [73]. Dysfunction of this compensatory capacity will play a role in the development of OA when the compensatory capacity of chondrocytes cannot adapt to the changing stress conditions of the body. Chondrocyte apoptosis is a part of the homeostasis of cartilage tissue under physiological conditions. However, the damaged OA chondrocytes enter the apoptosis process early, and secrete a variety of cytokines known as senescence-associated secretory phenotypes (SASP) or as extracellular signal modulators [74,75]. The SASP has been shown to induce elevated production of both local inflammatory factors and oxidative stress molecules, leading to heightened local or systemic inflammatory responses [74,75]. With the development of OA, the synthetic functions of articular chondrocytes gradually decrease while the level of apoptosis gradually increases.

Wang et al. showed that the apoptosis rate of knee joint chondrocytes in OA rabbits was significantly lower after the intervention of resveratrol (10, 20 or 50 μmol/kg/d for two consecutive weeks) [37]. Yuce et al. found that temporomandibular joint chondrocyte apoptosis was significantly inhibited after administration of resveratrol (100 μg/80 mL or 1 mg/80 mL for four consecutive weeks) in the temporomandibular joint (TMJ) of rats with TMJ OA [38]. At the same time, it was found that the apoptosis rate of chondrocytes in the high-dose resveratrol (1 mg/80 mL) group was lower than that in the low-dose resveratrol (100 μg/80 mL) group. After giving resveratrol (30, 60 or 120 mg/kg/d for six weeks) to OA rabbits, Wang et al. found that the apoptosis of knee joint chondrocytes was significantly inhibited [47]. A high dose of resveratrol (120 mg/kg/d) exerted a more significant inhibitory effect on the apoptosis of knee articular chondrocytes. The meta-analysis of this review found that resveratrol can inhibit chondrocyte apoptosis dose-dependently.

Resveratrol has been found to bind to several small molecules in various signalling pathways associated with inflammation [76,77]. Additionally, numerous studies reported that resveratrol acts as a potent SIRT1 agonist. The stimulation of SIRT1 activity by resveratrol could help in reducing inflammation [29,30]. SIRT1, a gene associated with longevity, has been implicated in many age-related diseases, including OA [[78], [79], [80], [81], [82]]. SIRT1 plays a crucial role in regulating the expression of extracellular matrix-associated proteins, promoting mesenchymal stem cell differentiation, and exerting anti-catabolic, anti-inflammatory, anti-oxidative stress, and anti-apoptotic effects [78]. Resveratrol can also regulate the nuclear factor kappa-B signalling pathway via SIRT1 to reduce inflammatory cell infiltration in the joint cavity of rheumatoid arthritis (RA) and/or OA while inhibiting synovial cell proliferation [30,83]. Resveratrol also inhibited IL-1β, TNF-α, and IL-6 production in RA synovial cells via the phosphoinositide-3-kinase (PI3K)/Akt signalling pathway [84]. Despite differing in the initial pathological changes, both OA and RA share a common feature of inflammatory cell infiltration, including macrophages and T cells. These infiltrating cells release a host of pro-inflammatory cytokines, such as IL-1β, TNF-α, and IL-6, which contribute to the amplification of the inflammatory response and tissue damage within the joint cavity [85,86]. In conclusion, resveratrol has demonstrated promising effects in preventing inflammatory responses in OA. However, its molecular mechanisms are complex and involve multiple signal transduction pathways, necessitating further in-depth investigation.

Resveratrol has been confirmed in multiple animal models to inhibit apoptosis, vasodilation, endothelial protection, and anti-atherosclerosis by preventing the cascade reaction of apoptosis [22,25]. Studies have shown that resveratrol can regulate chondrocyte metabolism, slow down chondrocyte ageing, reduce apoptosis and enhance chondrocyte autophagy through the SIRT1 family, and play a role in maintaining ECM homeostasis [87,88]. Additionally, resveratrol could target miRNAs associated with bacterial meningitis and reduce the apoptotic cell death index [77]. Emerging research indicates that the endoplasmic reticulum stress (ERS) response may contribute to developing degenerative diseases, such as OA, associated with ageing [89]. Resveratrol can alleviate ERS in various ways to inhibit chondrocyte apoptosis. These common pathways include increasing the folding and modification of endoplasmic reticulum proteins, regulating endoplasmic reticulum-related signalling pathways, and inhibiting the occurrence of related inflammatory responses and oxidative stress responses [90,91]. Combining the previous studies with this meta-analysis, resveratrol is shown to have a positive effect on reducing cellular apoptosis including OA articular chondrocytes. However, it involves a variety of genes and signal transduction pathways. To validate the effects of resveratrol in OA, the relevant molecular pathways still need to be verified.

The Mankin grading system is a standard method to assess the severity of damage to articular cartilage [[92], [93], [94]]. The meta-analysis of the six studies suggested that the Mankin scores of the experimental group were significantly improved compared to the control group, indicating that resveratrol exerted beneficial effects on the overall management of OA. Nevertheless, the Mankin grading system scores showed a high risk of publication bias. This may be due to our inclusion criteria where we only included published articles in Chinese and English language but not grey literature. Additionally, the subjective factors of researchers may affect the accuracy and reliability of the Mankin scores. Besides, a positive and promising finding is having a higher success rate in publication acceptance [95].

Previous studies demonstrated that resveratrol helped to prevent chronic diseases including cancer, cardiovascular disease, obesity and OA [23,25,30]. Safety studies on animals have shown that resveratrol supplementation possesses minimal adverse effects [96]. No deaths or abnormalities were reported in mice given an oral dose of 45 mg/kg/day resveratrol for 12 weeks [39]. Another study reported no toxic effects in rabbits given an oral dose of 120 mg/kg/day resveratrol for six weeks [38]. Several clinical trials have reported that resveratrol, or its trans stereoisomer, is generally safe with tolerable adverse effects [97]. Apart from that, OA is a degenerative disease affecting joint functions and structures. Despite the evidence on cartilage structural changes, few studies examined the changes in joint functions, such as grip strength, paw weight-bearing test and gait analysis. Few studies extended the scope examination beyond cartilage to other anatomical structures important for the pathogenesis of OA, such as the infrapatellar fat pad and subchondral bone. Ultimately, human clinical trials are still needed to validate the joint-protecting data of resveratrol derived from animal studies. We only found one clinical trial registered (ClinicalTrials.gov identifier: NCT02905799) to study the effects of resveratrol on osteoarthritic pain but the results have not been released [98].

This review is not without its limitations. Despite the positive findings on the joint protective effects of resveratrol, the overall robustness of the evidence is diminished due to the limited number of studies included and heterogeneity in study design. High variability and heterogenicity were reported in some parameters assessed, such as synovial and serum IL-6, synovial TNF-α and Mankin scores, contributing to lower power. Hence, the results for these indices should be interpreted with caution. This issue could only be resolved with a larger sample size. Although challenges remain in translating these preclinical findings to humans for the treatment of OA, it is noteworthy that human clinical trials involving resveratrol as a treatment for other diseases have been completed or are in development [99]. More well-designed preclinical studies of resveratrol-based therapies involving OA, limiting bias and ensuring safety checks for resveratrol use, are critical to advancing the use of resveratrol-based therapies in patients with this disease.

There are several limitations to this study. First, in all 15 studies, short-term treatment duration (≤12 weeks) was employed, and no study is available for medium- and long-term of treatment. Second, we focused only on animal studies, which may have potential confounding bias compared with clinical trials. This is parallel with the high risk of publication bias as demonstrated in Fig. 9. Third, we only searched for articles written in English and Chinese and did not include papers in other languages. Fourth, we only considered published articles and potential negative studies not published were not included.

## 5 Conclusion

OA is a disease with a high global prevalence, and the persistence of its chronic inflammation dramatically increases the risk of causing disability and leading to joint deformity. Resveratrol exerts therapeutic effects against OA through its anti-inflammatory, antioxidant, and anti-apoptotic effects on articular chondrocytes. Although the studies on resveratrol in this review consistently showed positive therapeutic effects on OA, the small number of studies included and the lower power of analysis in several parameters should be considered in interpreting the results of this meta-analysis. More in-depth preclinical studies on this topic will be needed to consolidate the findings, understand the molecular mechanism involved and pave the way for clinical trials on patients with OA.

## Funding

This project is funded by Fundamental Research Grant, Faculty of Medicine, 10.13039/501100004515Universiti Kebangsaan Malaysia (Code: FF-2022-400).

## Data availability

Data are available at reasonable request from the corresponding author.

## CRediT authorship contribution statement

**Wenjian Zhao:** Writing – original draft, Methodology, Investigation, Formal analysis, Conceptualization. **Yuezhi Zhu:** Writing – review & editing, Validation, Investigation. **Sok Kuan Wong:** Writing – review & editing, Supervision, Project administration. **Norliza Muhammad:** Writing – review & editing, Supervision, Project administration. **Kok-Lun Pang:** Writing – review & editing, Validation. **Kok-Yong Chin:** Writing – review & editing, Validation, Supervision, Project administration, Conceptualization.

## Declaration of competing interest

The authors declare that they have no known competing financial interests or personal relationships that could have appeared to influence the work reported in this paper.

## References

[1] Chen D., Shen J., Zhao W., Wang T., Han L., Hamilton J.L. (2017). Osteoarthritis: toward a comprehensive understanding of pathological mechanism. Bone Res..

[2] Duong V., Hunter D.J. (2023). Osteoarthritis research is failing to reach consumers. Nat. Rev. Rheumatol..

[3] Hsu H., Siwiec R.M., osteoarthritis Knee (2022). 2022 Sept 4 [cited 2023 July 17]. in: StatPearls [Internet] [Internet]. Treasure Island: StatPearls Publishing, [cited 2023 July 17].

[4] Huang C.H., James K., Lanois C., Corrigan P., Yen S.C., Stefanik J. (2023). Inter-joint coordination variability is associated with pain severity and joint loading in persons with knee osteoarthritis. J. Orthop. Res..

[5] Arden N., Nevitt M.C. (2006). Osteoarthritis: epidemiology. Best Pract. Res. Clin. Rheumatol..

[6] Prevention CfDCa (2021).

[7] Shalhoub M., Anaya M., Deek S., Zaben A.H., Abdalla M.A., Jaber M.M. (2022). The impact of pain on quality of life in patients with osteoarthritis: a cross-sectional study from Palestine. BMC Muscoskel. Disord..

[8] Farr Ii J., Miller L.E., Block J.E. (2013). Quality of life in patients with knee osteoarthritis: a commentary on nonsurgical and surgical treatments. Open Orthop. J..

[9] Liu M., Jin F., Yao X., Zhu Z. (2022). Disease burden of osteoarthritis of the knee and hip due to a high body mass index in China and the USA: 1990–2019 findings from the global burden of disease study 2019. BMC Muscoskel. Disord..

[10] Chow Y.Y., Chin K.Y. (2020). The role of inflammation in the pathogenesis of osteoarthritis. Mediat. Inflamm..

[11] Sokolove J., Lepus C.M. (2013). Role of inflammation in the pathogenesis of osteoarthritis: latest findings and interpretations. Ther Adv Musculoskelet Dis.

[12] Sanchez-Lopez E., Coras R., Torres A., Lane N.E., Guma M. (2022). Synovial inflammation in osteoarthritis progression. Nat. Rev. Rheumatol..

[13] Barker K.L., Toye F., Seers K. (2023). A synthesis of qualitative research to understand the complexity behind treatment decision-making for osteoarthritis. Osteoarthr Cartil Open.

[14] Yu S.P., Hunter D.J. (2015). Managing osteoarthritis. Aust. Prescr..

[15] Maqbool M., Fekadu G., Jiang X., Bekele F., Tolossa T., Turi E. (2021). An up to date on clinical prospects and management of osteoarthritis. Ann Med Surg.

[16] Gezer H.H., Ostor A. (2023). What is new in pharmacological treatment for osteoarthritis?. Best Pract. Res. Clin. Rheumatol..

[17] Maniar K.H., Jones I.A., Gopalakrishna R., Vangsness C.T. (2018). Lowering side effects of NSAID usage in osteoarthritis: recent attempts at minimizing dosage. Expet Opin. Pharmacother..

[18] Horl W.H. (2010). Nonsteroidal anti-inflammatory drugs and the kidney. Pharmaceuticals.

[19] Gunter B.R., Butler K.A., Wallace R.L., Smith S.M., Harirforoosh S. (2017). Non-steroidal anti-inflammatory drug-induced cardiovascular adverse events: a meta-analysis. J. Clin. Pharm. Therapeut..

[20] Navarro R.A., Adams A.L., Lin C.C., Fleming J., Garcia I.A., Lee J. (2020). Does knee arthroscopy for treatment of meniscal damage with osteoarthritis Delay knee replacement compared to physical therapy alone?. Clin. Orthop. Surg..

[21] Romina B.-P., Gordon H.G., Rachelle B., Rudolf W.P., Stefan S., Yaping C. (2017). Knee arthroscopy versus conservative management in patients with degenerative knee disease: a systematic review. BMJ Open.

[22] Koushki M., Amiri-Dashatan N., Ahmadi N., Abbaszadeh H.A., Rezaei-Tavirani M. (2018). Resveratrol: a miraculous natural compound for diseases treatment. Food Sci. Nutr..

[23] Shan G., Minchao K., Jizhao W., Rui Z., Guangjian Z., Jin Z. (2023). Resveratrol improves the cytotoxic effect of CD8 +T cells in the tumor microenvironment by regulating HMMR/Ferroptosis in lung squamous cell carcinoma. J. Pharm. Biomed. Anal..

[24] Wang C., Ye X., Ding C., Zhou M., Li W., Wang Y. (2023). Two resveratrol oligomers inhibit cathepsin L activity to suppress SARS-CoV-2 entry. J. Agric. Food Chem..

[25] Pannu N., Bhatnagar A. (2019). Resveratrol: from enhanced biosynthesis and bioavailability to multitargeting chronic diseases. Biomed. Pharmacother..

[26] Wahab A., Gao K., Jia C., Zhang F., Tian G., Murtaza G. (2017). Significance of resveratrol in clinical management of chronic diseases. Molecules.

[27] Liu F.C., Hung L.F., Wu W.L., Chang D.M., Huang C.Y., Lai J.H. (2010). Chondroprotective effects and mechanisms of resveratrol in advanced glycation end products-stimulated chondrocytes. Arthritis Res. Ther..

[28] Wei Y., Jia J., Jin X., Tong W., Tian H. (2018). Resveratrol ameliorates inflammatory damage and protects against osteoarthritis in a rat model of osteoarthritis. Mol. Med. Rep..

[29] Yang S., Sun M., Zhang X. (2022). Protective effect of resveratrol on knee osteoarthritis and its molecular mechanisms: a recent review in preclinical and clinical trials. Front. Pharmacol..

[30] Liang C., Xing H., Wang C., Xu X., Hao Y., Qiu B. (2023). Resveratrol improves the progression of osteoarthritis by regulating the SIRT1-FoxO1 pathway-mediated cholesterol metabolism. Mediat. Inflamm..

[31] Page M.J., Moher D., Bossuyt P.M., Boutron I., Hoffmann T.C., Mulrow C.D. (2021). PRISMA 2020 explanation and elaboration: updated guidance and exemplars for reporting systematic reviews. BMJ.

[32] Hooijmans C.R., Rovers M.M., de Vries R.B., Leenaars M., Ritskes-Hoitinga M., Langendam M.W. (2014). SYRCLE's risk of bias tool for animal studies. BMC Med. Res. Methodol..

[33] Review Manager (RevMan) [Computer program] (2020).

[34] Borenstein M., Hedges L., Rothstein H. (2007). Meta-analysis.

[35] Dekkers O.M. (2018). Meta‐analysis: key features, potentials and misunderstandings. Res Pract Thromb Haemost.

[36] Griffin J.W. (2021). Calculating statistical power for meta-analysis using metapower. The Quantitative Methods for Psychology.

[37] Wang J., Gao J.S., Chen J.W., Li F., Tian J. (2012). Effect of resveratrol on cartilage protection and apoptosis inhibition in experimental osteoarthritis of rabbit. Rheumatol. Int..

[38] Yuce P., Hosgor H., Rencber S.F., Yazir Y. (2021). Effects of intra-articular resveratrol injections on cartilage destruction and synovial inflammation in experimental temporomandibular joint osteoarthritis. J. Oral Maxillofac. Surg..

[39] Jiang M., Li X., Yu X., Liu X., Xu X., He J. (2017). Oral administration of resveratrol alleviates osteoarthritis pathology in C57BL/6J mice model induced by a high-fat diet. Mediat. Inflamm..

[40] Gu H., Li K., Li X., Yu X., Wang W., Ding L. (2016). Oral resveratrol prevents osteoarthritis progression in C57BL/6J mice fed a high-fat diet. Nutrients.

[41] Zhou Z., Deng Z., Liu Y., Zheng Y., Yang S., Lu W. (2021). Protective effect of SIRT1 activator on the knee with osteoarthritis. Front. Physiol..

[42] Wei Y., Jia J., Jin X., Tong W., Tian H. (2018). Resveratrol ameliorates inflammatory damage and protects against osteoarthritis in a rat model of osteoarthritis. Mol. Med. Rep..

[43] El-Bidawy M.H., Omar Hussain A.B., Al-Ghamdi S., Aldossari K.K., Haidara M.A., Al-Ani B. (2021). Resveratrol ameliorates type 2 diabetes mellitus-induced alterations to the knee joint articular cartilage ultrastructure in rats. Ultrastruct. Pathol..

[44] Xu X., Liu X., Yang Y., He J., Gu H., Jiang M. (2019). Resveratrol inhibits the development of obesity-related osteoarthritis via the TLR4 and PI3K/Akt signaling pathways. Connect. Tissue Res..

[45] Ebrahim H.A., Alzamil N.M., Al-Ani B., Haidara M.A., Kamar S.S., Dawood A.F. (2022). Suppression of knee joint osteoarthritis induced secondary to type 2 diabetes mellitus in rats by resveratrol: role of glycated haemoglobin and hyperlipidaemia and biomarkers of inflammation and oxidative stress. Arch. Physiol. Biochem..

[46] 童敏 高戈, 高洁生 向大雄 (2007). 白藜芦醇对骨关节炎模型兔关节液中一氧化氮水平的影响. 新乡医学院学报.

[47] 王静 高洁生, 陈进伟 李芬, 田静 谢希 (2009). 白藜芦醇对实验性兔骨关节炎软骨细胞凋亡及bcl-2, bax表达的影响. 中华风湿病学杂志.

[48] 高戈 徐克前, 田静 杜金峰, 谢希 高洁生 (2012). 白藜芦醇对兔实验性骨关节炎白细胞介素-1β表达的影响. 中国医院药学杂志.

[49] 陈蓟 雷鸣, 周震全 刘弼, 白藜芦醇对兔膝骨关节炎的保护作用 肖德明 (2017). 中国矫形外科杂志.

[50] 童敏 高戈, 高洁生 向大雄 (2007). 白藜芦醇甲基化衍生物对兔骨关节炎模型关节液及血清NO和iNOS水平的影响. 湖南中医药大学学报.

[51] 饶慧 高洁生, 高戈 童敏 (2008). 白藜芦醇抑制兔实验性骨关节炎血清白介素-6的研究. 中南药学.

[52] Tschon M., Contartese D., Pagani S., Borsari V., Fini M. (2021). Gender and sex are key determinants in osteoarthritis not only confounding variables. A systematic review of clinical data. J. Clin. Med..

[53] Loucks A., Maerz T., Hankenson K., Moeser A., Colbath A. (2023). The multifaceted role of mast cells in joint inflammation and arthritis. Osteoarthritis Cartilage.

[54] Zhang J.M., An J. (2007). Cytokines, inflammation, and pain. Int. Anesthesiol. Clin..

[55] Wong S.K., Chin K.Y., Ima-Nirwana S. (2019). Toll-like receptor as a molecular link between metabolic syndrome and inflammation: a review. Curr. Drug Targets.

[56] Jrad A.I.S., Trad M., Bzeih W., El Hasbani G., Uthman I. (2023). Role of pro-inflammatory interleukins in osteoarthritis: a narrative review. Connect. Tissue Res..

[57] Vincent T.L. (2019). IL-1 in osteoarthritis: time for a critical review of the literature. F1000Res.

[58] Melo A. (2011). IL-1 and its role in osteoarthritis. Open J Med.

[59] Wang B., Bellot G.L., Iskandar K., Chong T.W., Goh F.Y., Tai J.J. (2020). Resveratrol attenuates TLR-4 mediated inflammation and elicits therapeutic potential in models of sepsis. Sci. Rep..

[60] Jakus P.B., Kalman N., Antus C., Radnai B., Tucsek Z., Gallyas F. (2013). TRAF6 is functional in inhibition of TLR4-mediated NF-κB activation by resveratrol. J. Nutr. Biochem..

[61] Zhou Q., Ren Q., Jiao L., Huang J., Yi J., Chen J. (2022). The potential roles of JAK/STAT signaling in the progression of osteoarthritis. Front. Endocrinol..

[62] Augustin L., Chahrazad C., Jérémy M., Hang-Korng E., Wafa B., Thomas F.-B. (2017). Systemic inhibition of IL-6/Stat3 signalling protects against experimental osteoarthritis. Ann. Rheum. Dis..

[63] Chen B., Ning K., Sun M.L., Zhang X.A. (2023). Regulation and therapy, the role of JAK2/STAT3 signaling pathway in OA: a systematic review. Cell Commun. Signal..

[64] Keller Laura E., Tait Wojno Elia D., Begum L., Fortier Lisa A. (2022). Interleukin-6 neutralization and Regulatory T cells are additive in chondroprotection from IL-1ß-induced inflammation. J. Orthop. Res..

[65] Luo W., Zhang G., Wang Z., Wu Y., Xiong Y. (2023). Ubiquitin-specific proteases: vital regulatory molecules in bone and bone-related diseases. Int Immunopharmacol.

[66] Chisari E., Yaghmour K.M., Khan W.S. (2020). The effects of TNF-alpha inhibition on cartilage: a systematic review of preclinical studies. Osteoarthritis Cartilage.

[67] Guo Y., Li R., Dang X. (2022). S100A10 regulates tumor necrosis factor alpha-induced apoptosis in chondrocytes via the reactive oxygen species/nuclear factor-kappa B pathway. Biotechnol. Appl. Biochem..

[68] Zhu H., Li X., Qiao M., Sun X., Li G. (2023). Resveratrol alleviates inflammation and ER stress through SIRT1/NRF2 to delay ovarian aging in a short-lived fish. J Gerontol A Biol Sci Med Sci.

[69] Cinelli M.A., Do H.T., Miley G.P., Silverman R.B. (2020). Inducible nitric oxide synthase: regulation, structure, and inhibition. Med. Res. Rev..

[70] Ahmad N., Ansari M.Y., Haqqi T.M. (2020). Role of iNOS in osteoarthritis: pathological and therapeutic aspects. J. Cell. Physiol..

[71] Jiang H., Ji P., Shang X., Zhou Y. (2023). Connection between osteoarthritis and nitric oxide: from pathophysiology to therapeutic target. Molecules.

[72] Alavi M., Farkhondeh T., Aschner M., Samarghandian S. (2021). Resveratrol mediates its anti-cancer effects by Nrf2 signaling pathway activation. Cancer Cell Int..

[73] Zheng L., Zhang Z., Sheng P., Mobasheri A. (2021). The role of metabolism in chondrocyte dysfunction and the progression of osteoarthritis. Ageing Res. Rev..

[74] Wu C.J., Liu R.X., Huan S.W., Tang W., Zeng Y.K., Zhang J.C. (2022). Senescent skeletal cells cross-talk with synovial cells plays a key role in the pathogenesis of osteoarthritis. Arthritis Res. Ther..

[75] Loeser R.F., Collins J.A., Diekman B.O. (2016). Ageing and the pathogenesis of osteoarthritis. Nat. Rev. Rheumatol..

[76] Zhao Y., Li Z., Wang X., Zhao F., Wang C., Zhang Q. (2022). Resveratrol attenuates heat stress-induced impairment of meat quality in broilers by regulating the Nrf2 signaling pathway. Animals.

[77] de Queiroz K.B., Dos Santos Fontes Pereira T., Araújo M.S.S., Gomez R.S., Coimbra R.S. (2018). Resveratrol acts anti-inflammatory and neuroprotective in an infant rat model of pneumococcal meningitis by modulating the hippocampal miRNome. Mol. Neurobiol..

[78] Yang Y., Liu Y., Wang Y., Chao Y., Zhang J., Jia Y. (2022). Regulation of SIRT1 and its roles in inflammation. Front. Immunol..

[79] Rahman S., Islam R. (2011). Mammalian Sirt1: insights on its biological functions. Cell Commun. Signal..

[80] Elibol B., Kilic U. (2018). High levels of SIRT1 expression as a protective mechanism against disease-related conditions. Front. Endocrinol..

[81] Sun K., Wu Y., Zeng Y., Xu J., Wu L., Li M. (2022). The role of the sirtuin family in cartilage and osteoarthritis: molecular mechanisms and therapeutic targets. Arthritis Res. Ther..

[82] Chin K.-Y., Ekeuku S.O., Pang K.-L. (2022). Sclerostin in the development of osteoarthritis: a mini review. Malays. J. Pathol..

[83] Sheng S., Wang X., Liu X., Hu X., Shao Y., Wang G. (2022). The role of resveratrol on rheumatoid arthritis: from bench to bedside. Front. Pharmacol..

[84] Tian J., Chen J.W., Gao J.S., Li L., Xie X. (2013). Resveratrol inhibits TNF-α-induced IL-1β, MMP-3 production in human rheumatoid arthritis fibroblast-like synoviocytes via modulation of PI3kinase/Akt pathway. Rheumatol. Int..

[85] Chen Y., Jiang W., Yong H., He M., Yang Y., Deng Z. (2020). Macrophages in osteoarthritis: pathophysiology and therapeutics. Am J Transl Res.

[86] Xu Y., Zhang Z., He J., Chen Z. (2022). Immune effects of macrophages in rheumatoid arthritis: a bibliometric analysis from 2000 to 2021. Front. Immunol..

[87] Kim H.J., Braun H.J., Dragoo J.L. (2014). The effect of resveratrol on normal and osteoarthritic chondrocyte metabolism. Bone Joint Res.

[88] Liang C., Xing H., Wang C., Xu X., Hao Y., Qiu B. (2022). Resveratrol protection against IL-1β-induced chondrocyte damage via the SIRT1/FOXO1 signaling pathway. J. Orthop. Surg. Res..

[89] Wen Z., Sun Q., Shan Y., Xie W., Ding Y., Wang W. (2023). Endoplasmic reticulum stress in osteoarthritis: a novel perspective on the pathogenesis and treatment. Aging Dis.

[90] Yin Y., Lv G., Zhang W., Yuan J., Yang Y., Wang Y. (2023). Resveratrol glycoside mediates microglial endoplasmic reticulum stress to mitigate LPS-induced sepsis-associated cognitive dysfunction. Behav. Brain Res..

[91] Önay Uçar E., Sengelen A., Mertoglu Kamali E. (2023). Hsp27, Hsp60, Hsp70, or Hsp90 depletion enhances the antitumor effects of resveratrol via oxidative and ER stress response in human glioblastoma cells. Biochem. Pharmacol..

[92] Mantripragada V.P., Bova W.A., Boehm C., Piuzzi N.S., Obuchowski N.A., Midura R.J. (2018). Progenitor cells from different zones of human cartilage and their correlation with histopathological osteoarthritis progression. J. Orthop. Res..

[93] Pearson R.G., Kurien T., Shu K.S.S., Scammell B.E. (2011). Histopathology grading systems for characterisation of human knee osteoarthritis – reproducibility, variability, reliability, correlation, and validity. Osteoarthritis Cartilage.

[94] Al-Saadi H.M., Chin K.-Y., Ahmad F., Mohd Ramli E.S., Arlamsyah A.M., Japar Sidik F.Z. (2021). Effects of palm tocotrienol-rich fraction alone or in combination with glucosamine sulphate on grip strength, cartilage structure and joint remodelling markers in a rat model of osteoarthritis. Appl. Sci..

[95] Mlinaric A., Horvat M., Šupak Smolcic V. (2017). Dealing with the positive publication bias: why you should really publish your negative results. Biochem. Med..

[96] Shaito A., Posadino A.M., Younes N., Hasan H., Halabi S., Alhababi D. (2020). Potential adverse effects of resveratrol: a literature review. Int. J. Mol. Sci..

[97] Ramírez-Garza S.L., Laveriano-Santos E.P., Marhuenda-Muñoz M., Storniolo C.E., Tresserra-Rimbau A., Vallverdú-Queralt A. (2018). Health effects of resveratrol: results from human intervention trials. Nutrients.

[98] gov ClinicalTrials (2022). https://classic.clinicaltrials.gov/ct2/show/NCT02905799.

[99] Berman A.Y., Motechin R.A., Wiesenfeld M.Y., Holz M.K. (2017). The therapeutic potential of resveratrol: a review of clinical trials. npj Precision Onc.

